# Effects of prebiotics on microbial diversity and abundance in young children with acute malnutrition: study protocol for a multi-centered, double-blinded randomized controlled trial

**DOI:** 10.1186/s13063-024-08647-z

**Published:** 2024-11-26

**Authors:** Javeria Saleem, Rubeena Zakar, Sanaullah Iqbal, Muhammad Arshad, Ruhma Shahzad, Munazza Batool, Muhammad Nawaz, Muhammad Salman Butt, Florian Fischer

**Affiliations:** 1 Department of Public Health, University of the Punjab, Lahore, Pakistan; 2 Department of Food Science and Human Nutrition, University of Veterinary and Animal Sciences, Lahore, Pakistan; 3 Centre for Genomics and Systems Biology, New York University Abu Dhabi, Abu Dhabi, United Arab Emirates; 4 Institute of Microbiology, University of Veterinary and Animal Sciences, Lahore, Pakistan; 5 Faculty of Life and Health Sciences, Ulster University, Belfast, UK; 6 Institute of Public Health, Charité - Universitätsmedizin Berlin, Berlin, Germany

**Keywords:** RUTF, Prebiotics, SAM, Gut microbiome

## Abstract

**Background:**

The anti-inflammatory and antimicrobial benefits of prebiotics may present an affordable and cost-effective strategy for not only the prevention but also treatment of malnutrition. Therefore, the present trial has been designed with the aim to evaluate the role of prebiotics on the gut microbiome of severe acute malnourished (SAM) children.

**Methods:**

The study is designed as a prospective, double-blinded, triple-armed, multi-centered randomized controlled trial, with 6–59 months old uncomplicated SAM children recruited to the experimental group receiving ready-to-use therapeutic food (RUTF) plus prebiotics and the active comparator group receiving RUTF plus starch for 2 months duration (8 weeks). Healthy children with matching age and gender will be recruited to placebo comparator group and will receive starch as a placebo during the study period. A total of 58 participants will be recruited to each arm with 1:1:1 allocation ratio following a pre-defined inclusion and exclusion criteria. The results of the gut microbiome diversity will serve as the primary outcome, while weight-for-height/length *z*-score, mid-upper-arm circumference, neurodevelopment assessment, and body mass accumulation will serve as the secondary outcome. Data collection and evaluations will be conducted at baseline and at the end of the trial (week 8), while the safety monitoring will be conducted at every second week. For analysis, the principles of intention-to-treat will be followed.

**Conclusions:**

Conclusively, the results of the present trial would provide useful insights and high-quality data for the treatment and management of SAM children by evaluating the effect of RUTF plus prebiotic on the gut microbiome diversity of children, leading to medical evidence for designing the large-scale studies.

**Trial registration:**

The present trial is registered at ClinicalTrials.gov with identifier No: NCT06155474 and registration date 4 December 2023.

## Background

Malnutrition is a major global public health concern with its outcomes linked to poor health and early morbidities among children under five, particularly in developing countries. Pakistan reported to have one of the highest prevalence of malnutrition among children in comparison to other developing countries [1]. About 45% of children are projected to be stunted, 10.5% wasted, and 31.6% underweight [2]. In 2021, 5 million children died worldwide due to infectious diseases or malnutrition, with Pakistan having the highest under-five mortality rate [3]. With a population of approximately 229 million, Pakistan is reported to be the fifth most populous country in the world. The under-five mortality in the country has reported to be 63.3 per 1000 live births in 2021 [3, 4]. Although mortality and malnutrition are often attributed to infections among under-five children, latest research highlights the presence of immature gut microbiome among malnourished children [5–9].

Gut microbiome are distinct microbial communities where trillions of different microscopic organisms including viruses, bacteria, and fungi live [10]. These microscopic organisms play a significant part in strengthening the gut integrity [11], regulating immunity [12], protecting against pathogens [13], harvesting energy [14], and influencing functions beyond the gut [10]. Nevertheless, the impairments in gut microbiome can be triggered by various environmental and subclinical characteristics that could lead to reduced intelligence quotient, growth faltering, and severely acute malnutrition (SAM) among children [9, 15–17].

With persistence iron and B_12_ deficiency and symptoms of diarrhea, pneumonia, acute gastroenteritis, and acute respiratory infections among SAM children [18], ready-to-use therapeutic food (RUTF) has proven to be a revolutionary rehabilitative therapy. The therapy of RUTF is used for managing the malnutrition among children by promoting weight gain and improving developmental potential [19, 20]. However, iron present in standard RUTF may not be sufficient to elevate circulating levels of ferritin into the optimal physiological range as one meta-analysis revealed higher hemoglobin levels and lower rates of anemia among SAM children when treated with high iron content as compared to standard RUTF [21]. The rate of relapse has also been a question among children treated with RUTF.

With deficiency of iron recognized in malnourished children [18], galactooligosaccharides (GOS), a form of prebiotic, have been proven to have anti-inflammatory and antimicrobial actions that might enhance response to standard therapy for SAM [22, 23]. These benefits have developed a rising interest in the use of prebiotics in diet to adjust gut microbiome in a way that can improve microbial health [24]. Targeting the gut microbiota through the use of prebiotics as a part of treatment in malnourished children may present an affordable and cost-effective strategy for the prevention and treatment of malnutrition among children as well as in improving their overall health [22]. Prebiotics are considered as a fuel for probiotics bacteria of gut that could protect the host against diarrhea with reduced rates of relapse. However, randomized controlled trials evaluating the effects of prebiotic supplementation on the gut microbiome among children with SAM have not previously been studied. So, there is a gap, as clinical evidence is lacking and the existing knowledge cannot be translated into clinical practice.

Around one trillion organisms per milliliter of luminal contents are found in the distal intestine, where the majority of these bacteria are found [10]. It is essential to describe the microbiota in malnourished children in order to define their potential to harvest energy and nutrients [25]. Therefore, the present study has been designed with the goal to evaluate the effect of prebiotics on the gut microbiome diversity in malnutrition children utilizing a prospective, double-blinded, triple-armed, multi-centered randomized controlled trial. The specific objectives of the present study are to:

- Examine the role of prebiotics on the microbiome diversity and abundance among malnourished children using 16 Svedberg (S) ribosomal ribonucleic acid gene sequencing
- Investigate the role of prebiotics on the anthropometric measures and the body composition of malnourished children
- Assess the impact of prebiotic supplementation on malnourished children’s neurodevelopment

## Methods

### Trial design

**Fig. 1:**
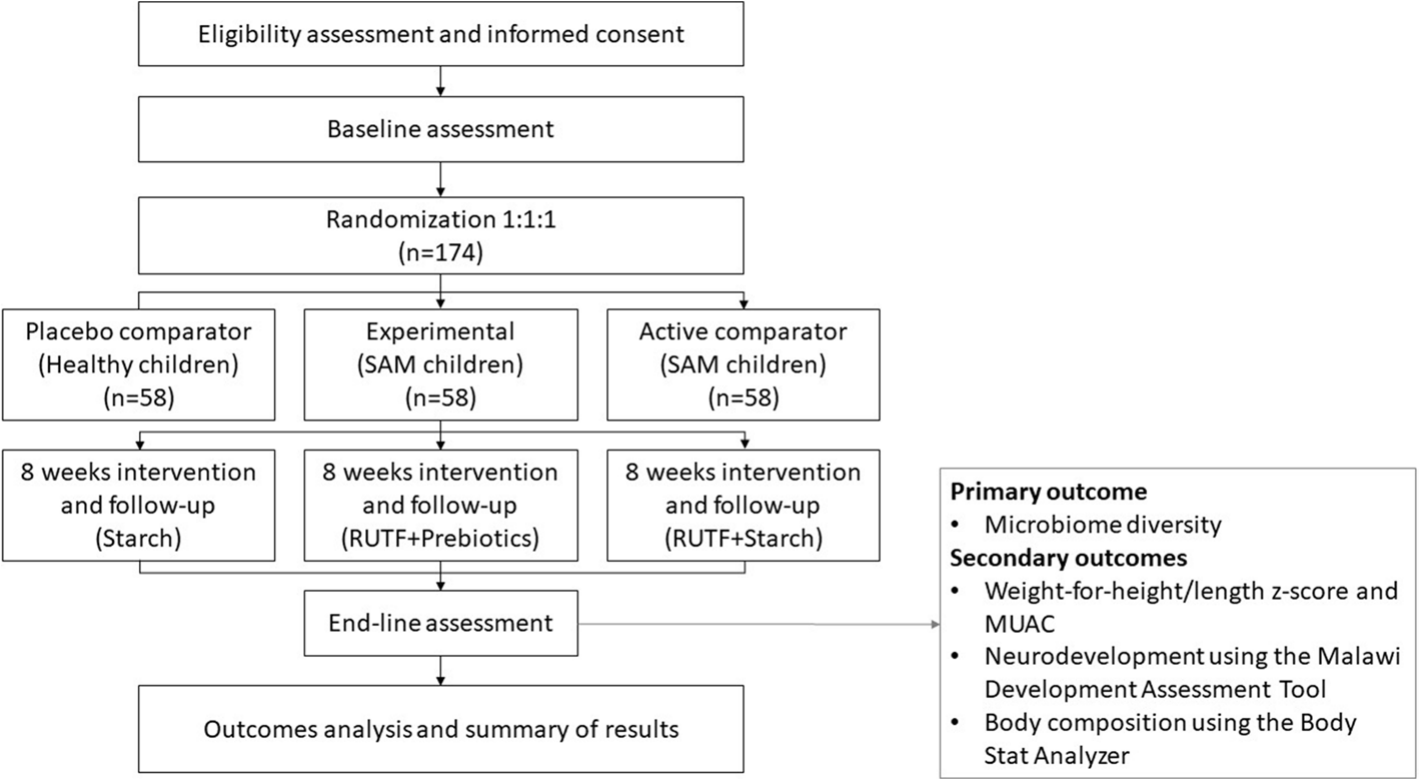
CONSORT flowchart

The present trial is designed as a prospective, double-blinded, triple-armed, multi-centered randomized controlled trial. The trial will be conducted following the guidelines Declaration of Helsinki, Standard Protocol Items: Recommendations for Interventional Trials (SPIRIT) [26], and Consolidated Standards of Reporting Trials (CONSORT) [27]. The trial design has been approved by the Institutional Ethics Review Board (IERB) (Letter No. D/137/FIMS) and is registered under the Clinical Trial Registry at www.clinicaltrials.gov with study ID NCT06155474. The flow diagram of the trail design is illustrated in Fig. 1.

### Study setting

The trial will be conducted at two public sector District Health Quarter (DHQ) level hospitals in Lahore, i.e., Government Mian Mir DHQ and Government Samnabad DHQ. Permissions will be taken from the relevant authorities of the hospitals. These two hospitals are selected as currently Integrated Reproductive Maternal Newborn, Child Health (IRMNCH) & Nutrition Programs are running in these hospitals with a high patient load.

### Study population and eligibility criteria

All children suffering from SAM, without any complications, and aged 6–59 months, are eligible for inclusion in the present trial. Participants will be screened using weight-for-height/length *z*-score and mid-upper arm circumference (MUAC) as screening criteria. The criteria of the World Health Organization (WHO) will be used to diagnose edema among children. WHO defines children with grade 1–2 bilateral edema and a weight-for-height/length *z*-score of < − 3 standard deviations (SD) or MUAC of < 11.5 cm who are otherwise clinically healthy, aware, and with a decent appetite as children with SAM [28]. Trained healthcare staff including nurse, childcare worker, and doctor will assess the children for eligibility. MUAC will be assessed using WHO’s MUAC colored tapes in centimeters (cm), while weight-for-height/length *z*-score and weight of children will be assessed in grams (g) with minimal and light clothing using UNISCALE, and height will be measured using a standard measuring tape in cm(s) and then will be compared with the grading of *z*-score chart. All the anthropometric measures will be taken thrice to reduce information bias. Additionally, all the instruments that will be used for taking measurements will be checked for accuracy and zero precision beforehand. All eligible participants will be recruited following a pre-defined inclusion and exclusion criteria (Table 1).

**Table 1:** Inclusion and exclusion criteria

*Inclusion criteria*	*Exclusion criteria*
Ages 6–59 months at enrolment	
Acute malnutrition without complications, as defined by the WHO (i.e., children with MUAC < 115 mm or weight-for-height *z*-score < − 3 or grade 1–2 bilateral edema who will be clinically well and alert with good appetite	Presence of any complications of severe malnutrition (severe dehydration, severe anemia, severe pitting edema, anorexia, hypothermia, high pyrexia, acute lower respiratory infection, or hypoglycemia) or immune compromised
Parental consent for the child to participate	Declined parental consent
Healthy children without any chronic or acute illness	Children on antibiotic treatment for any disease in the last 3 months

### Sample size estimation

**Fig. 2:**
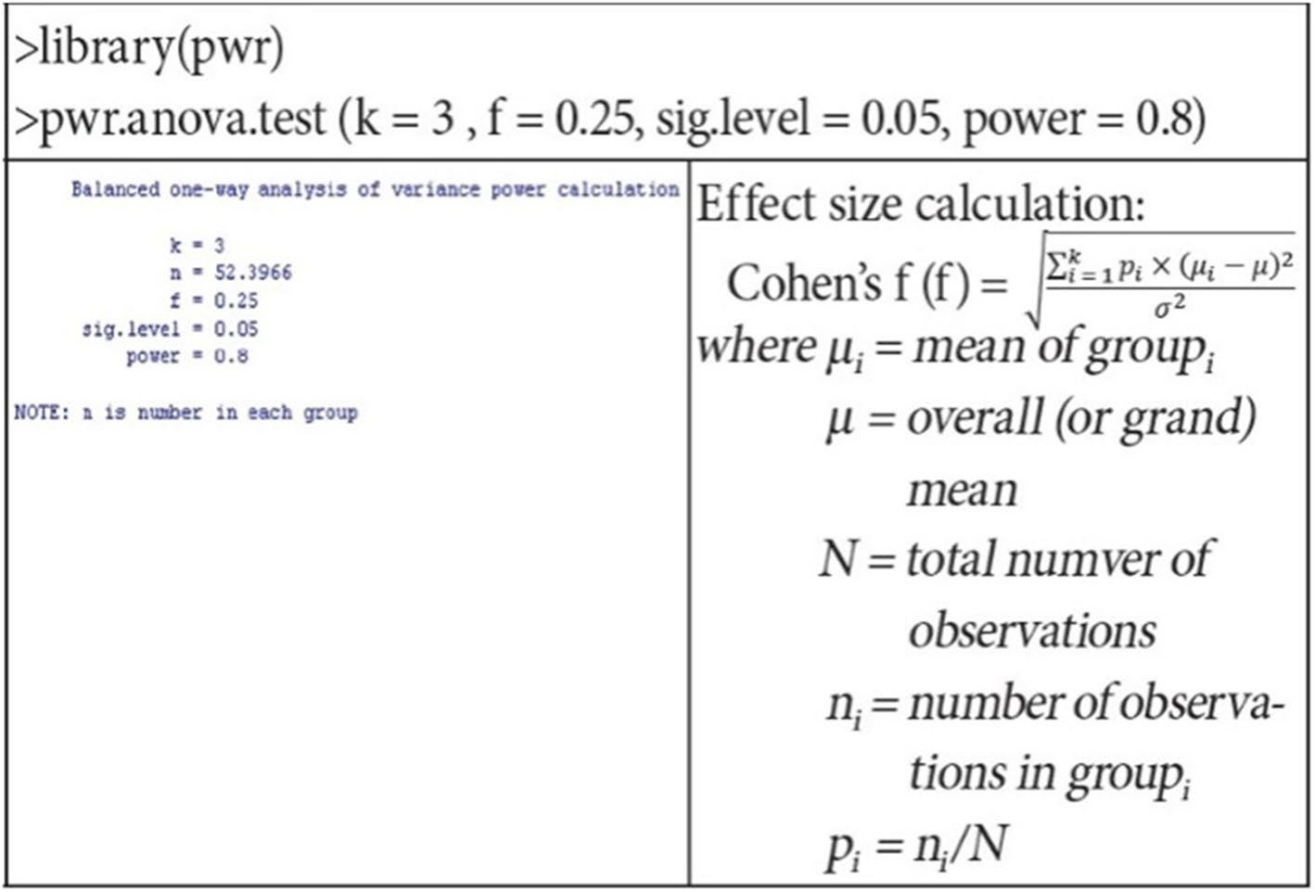
Sample calculation using R software

For sample size estimation, a balanced one-way analysis of variance power calculation with R software was used, and a minimum of sample of 53 participants was calculated for each group using 5% significance level, 80% power of study, 0.25 effect size (*f*) and 3 as *k* value (Fig. 2).

A calculated sample of 159 participants (53 participants each arm) is further inflated by 10% to account for attrition due to loss to follow-up, making a total sample of 174 participants with 58 participants required to be recruited in each arm. For achieving adequate participant enrolment to reach target sample size, community mobilization in the nearby areas of the selected hospitals through lady health workers will be carried out with the support of IRMNCH & Nutrition Program.

### Informed consent

Informed consent will be taken from the parents/guardians of the children on a written consent form. The purpose of the trial and the potential adverse reactions (i.e., allergies) of the treatment modalities will be clearly communicated to them. Upon their approval, thumb impression will be taken on the written consent form. The process of taking consent from each parent/guardian will be conducted in the presence of at least one witness.

### Randomization and allocation

Children suffering from acute malnutrition, otherwise healthy, aged 6–59 months, and fulfilling the inclusion criteria will be recruited randomly to one of the two arms of the present trial for 8 weeks, i.e., experimental and active comparator groups. However, healthy children with matched sex and age will be recruited from the vaccination centers of the research sites to the placebo comparator group as healthy controls. The participants in all groups will be recruited in equal allocation ratio (1:1:1) with no restriction of block size. A statistician unaware of the study method will generate the random numbers and allocate the participants to study groups by assigning unique code to them (i.e., SAM children to experimental and active comparator group and healthy children to placebo comparator group). All the participants will be given a recruitment card with their unique identity code mentioned on it.

### Blinding

A statistician unaware of the study, responsible for designating codes to the participants and allocation of groups, will prepare the packets of treatment regime for all the participants with day-wise dosage enclosed in separate opaque envelops and the unique code of the participants mentioned on it, blinding the participants and the investigator of the study. The treatment to all the participants will be given in separate rooms, and the parents of all the participants will be advised not to acquainted and communicate with each other after randomization for reducing the subjective biases. Blinding will only be permissible in case of withdrawal or termination of any study participants.

### Interventions

The detailed dietary intervention that will be provided to the participants recruited to each group is given in Table 2.

**Table 2:** Intervention for study groups

*Participant group/arm*	*Intervention/treatment*
Placebo comparator: healthy controls (starch) The healthy controls will be given starch packed in the same way as the active prebiotics	**Dietary supplement: starch only** The starch solution is a low-fat diet **Dosage:** A daily dosage of 4 g of starch mixed with water or milk
Experimental: malnutrition (RUTF plus prebiotics) The malnutrition group with RUTF and prebiotic only. The RUTF will be given as per the WHO guidelines	**Dietary supplement: prebiotic galactooligosaccharide (GOS) Powder** Prebiotic GOS powder is a galactooligosaccharide ingredient low in mono-sugars. Scientific studies have shown positive effects of oligosaccharides, among which GOS, on growth of bifidobacterial, stool consistency bowel function and transit time, support of natural defenses and mineral absorption The product mainly consists of 97% dry matter with 69% prebiotic GOS, 23% lactose, and 5% glucose and galactose as monosaccharides. These prebiotics have β-(4) glycosidic linkages and have been proven as safe product **Dosage:** In this arm, participants will receive a daily dosage of 4 g of prebiotic GOS mixed with milk or water along with RUTF. The dosage of RUTF will be determined based on the weight of the child in such a way that two sachets will be given to a child with a weight of 5–6.9 kg in a day/week, three sachets to a child weighting 7–9.9 kg in a day/week, and four sachets were given to a child with having weight less than or equal to 10 kg in a day/week
Active comparator: malnutrition (RUTF plus starch) The malnutrition group with RUTF and starch only. The RUTF will be given as per the WHO guidelines	**Dietary supplement: RUTF plus starch** RUTF and the starch solution **Dosage:** In this intervention arm of the trial, participants will receive a daily dose of 4 g of starch mixed with milk or water along with RUTF. The dosage of RUTF will be determined based on the weight of the child in such a way that two sachets will be given to a child with a weight of 5–6.9 kg in a day/week, three sachets to a child weighting 7–9.9 kg in a day/week, and four sachets were given to a child with having weight less than or equal to 10 kg in a day/week

### Withdrawal and termination criteria and management

RUTF will be provided as per the guidelines of WHO and all the therapies allowed by WHO will be permitted during the trial. However, children showing poor compliance or absence from two consecutive follow-up visits will be withdrawn from the study, while those showing any allergies or adverse events will be terminated from the study, and appropriate treatment will be provided to them after proper investigation by the principal investigator.

### Adverse events and safety monitoring

Throughout the trial, participants will be monitored on weekly basis to ensure safety. In case of any allergic reactions or adverse events, prognostic treatment will be provided after proper investigation free-of-cost. Further, participants will be encouraged to report to and contact the designated personnel in case of any discomfort or adverse event 24/7 to ensure the reporting and treatment of adverse events within 24 h. The principal investigator will then decide the continuation of the trial treatment after excluding for the factor unrelated to the trial medications. There are no anticipated harms of the interventions of the present trial; therefore, no post-trial care and compensation has been planned for participation in the present trial.

### Strategies for improving adherence

For improving the adherence of the trial, contact numbers of the parents of the participants will be collected, and a daily reminder text/call will be dropped. Empty treatment packets will not be collected for ensuring the blinding of the study. In addition to this, the parents of the participants will be incentivized through multi-vitamins for their other children and family members.

### Outcome parameters

All the assessments will be carried out by a trained staff unrelated to the enrolment and allocation procedures. The primary outcome of the trial is to examine the microbiome diversity of malnourished children, to compare it with healthy children, and to evaluate the effect of RUTF plus prebiotics on the microbiome diversity of malnourished children (Table 3). For achieving the primary outcome, 5 g stool sample of the children will be collected for DNA extraction using QIAamp DNA Stool kit (Qiagen, Hilden, Germany) for examining the microbiome diversity through 16 Svedberg (S) ribosomal ribonucleic acid gene sequencing.

**Table 3:** Primary outcome parameter

*Outcome measure*	*Measure description*	*Time frame*
Microbiome diversity	Microbiome diversity examination among malnourished children using 16 Svedberg (S) ribosomal ribonucleic acid gene sequencing. The microbiome diversity index measures the diversity of microbial species in malnourished children’s gut microbiota, comparing it to reference and control populations. Higher values indicate greater diversity, providing insights into malnutrition’s impact on gut microbiota composition	8 weeks

For the accomplishment of secondary outcome parameters, anthropometric measures of the children, including their height, weight (for calculating weight-for-height/length z-score), and MUAC will be assessed by a trained healthcare staff. For assessing neurodevelopment of children, the Malawi Development Assessment Tool (MDAT) will be used that is considered to be one of the most valid tools for assessing child development for ages 0–59 months measuring their motor, language, social, and cognitive development [29], while for assessing the muscle mass accumulation, Body Stat Analyzer will be used (Table 4).

**Table 4:** Secondary outcome parameters

*Outcome measures*	*Measure description*	*Time frame*
Anthropometric measurements	Change in the mean weight-for-height/length *z*-score and MUAC in children with complicated severe acute malnutrition who have been taking prebiotics supplementation for 8 weeks	8 weeks
Neurodevelopment assessment	Impact assessment of prebiotic supplementation on children’s neurodevelopment using the Malawi Development Assessment Tool	8 weeks
Muscle mass accumulation	The study investigates the impact of prebiotic consumption on body composition changes using the Body Stat Analyzer as a quantitative measurement tool	8 weeks

### Data collection

At baseline, sociodemographic characteristics of children including their age, gender, ethnicity, breast feeding history, mother’s demographics, mother’s nutritional status, and dietary habits will be recorded. The age of the child and the mother will be recorded through their national birth identity document, while the rest of the sociodemographic characteristics will be recorded through history of mother/parent/guardian. In addition to this, child’s anthropometric verbal measures (height, weight, and MUAC), neurodevelopment using MDAT, and muscle mass accumulation using body stat analyzer will be assessed, and 5 g stool sample of the children will be collected at baseline (week 0) and at the end of the trial (week 8) (Table 5). For collecting stool samples, stool samples kit will be used following the stool sample collection guidelines. Stool sample kits include a cardboard tube with inner metal container with one plastic container of preservation gel in it. For children using toilet seat, parents will be provided with a cardboard-and-tissue paper lining for fixing on the toilet seat, paper bowl for placing above the lining, disposable spoon for scooping the stool sample, and a sample collection tube for storing the sample. Parents will be instructed to ask the child to pass the urine before defecating the stool for ensuring that the sample is not contaminated with the urine and scoop out two spoons of the stool into the container. While for those children defecating in diapers, parents will be provided with one plastic wrap and will be instructed to place it in the diaper such that urine goes straight into diaper while the stool remains on the plastic wrap and to scoop out a sufficient amount into the container. All parents will be instructed to bring the sample the same day it has been collected. The samples will be labeled with the allocated unique identity codes and will be stored in a refrigerator at − 80 °C, while the rest of the data will be recorded using a structured performance by a trained healthcare staff. The timeline of the study is given in Table 5.

**Table 5:** Study timeline

	Enrollment/allocation	Intervention, follow-up, and monitoring								Close-out
Time point (week)	**0**	**1**	**2**	**3**	**4**	**5**	**6**	**7**	**8**	** > 8**
*Enrollment*										
Eligibility screening	x									
Informed consent	x									
Allocation		x								
*Interventions*										
Starch to placebo comparator group		x	x	x	x	x	x	x	x	
RUTF plus starch to active comparator group		x	x	x	x	x	x	x	x	
RUTF plus prebiotics to experimental group		x	x	x	x	x	x	x	x	
*Assessments*										
Sociodemographic characteristics	x									
Anthropometric measures (weight, height, MUAC)	x								x	
Fecal samples	x								x	
Malawi Developmental Assessment Tool	x								x	
Body stat analyzer	x								x	
Outcome analysis										x

### Statistical analysis

Intention-to-treat (ITT) analysis will be used in the present trial for analyzing the results. All the participants who are randomized, received at least one intervention, and evaluated for at least one outcome will be analyzed with missing data substituted according to the principle of ITT.

For evaluating the microbiome diversity, Ribosomal Database Project (RDP) Bayesian algorithm will be used to identify microbial structure at phylum and genus level among samples and trial groups using Fast Length Adjustment of Short Reads (FLASH) version 1.2.11 and software R version 3.4.1.

Raw sequence data will be filtered to obtain high-quality clean data with DADA2, after which clean reads that overlapped each other will be merged into tags and clustered to operational taxonomic units (OTU). Taxonomic classifications will be assigned to OTU representative sequences using the RDP database. FLASH version 1.2.11 will be used for generating consensus sequences for paired-end reads, and software R version 3.4.1 will be used to create phylum and genus abundance bar plots. Further, alpha diversity analysis will also be conducted. Alpha diversity is the analysis of species diversity in a sample, measured by observed species index, Chao1 index, Shannon index, Simpson index, and good-coverage index.

Furthermore, Statistical Package for Social Science (SPSS) version 24 will be used for evaluating the change in weight-to-height/length *z*-score, MUAC, neurodevelopment assessment, and muscle mass accumulation among trial participants. Firstly, for descriptive analysis, percentages and frequencies will be presented of qualitative variables (gender of the child, ethnicity, breast feeding history, and mother’s dietary habits), while mean ± SD (in case of normally distributed variables) and median ± inter-quartile range (IQR) (in case of skewed variables) will be given for quantitative variables. Furthermore, the association of the sociodemographic characteristics of the children and mothers with the weight-to-height/length z-score, MUAC, neurodevelopment assessment, and muscle mass accumulation among children will be explored through regression analysis. For comparing the outcomes between three trial groups, test of analysis of variance (ANOVA) will be used with Scheffe and Games-Howell test for post hoc multiple comparisons in case of normally distributed data; otherwise, Kruskal–Wallis test will be applied, while for analyzing the data of repeated measures within the same groups, paired-sample *t*-test (for normally distributed datasets) or will Wilcoxon test (for non-normally distributed datasets) will be applied. All the tests will be applied at 95% confidence interval (CI) by an independent statistician blinded to the study.

### Interim and additional analyses

No interim or temporary analysis has been planned for the present trial, as there are no anticipated harms of the interventions of the present trial.

### Quality control

For ensuring the quality of the data and trial, the trial steering committee, an Independent Data Monitoring Committee (IDMC) comprises a group of experts who are external to the clinical trial, and all the staff administered in the trial will be responsible. The administered trial staff responsible for randomization, allocation, data collection, and treatment administration will be provided with an extensive 2-day training by the principal investigator (first and second authors) before the initiation of the trial for introducing them to the specifics of the trial. Safety monitoring will be the responsibility of the Institutional Review Board (IRB) of University of the Punjab, who can inspect the records, documents, and assessors of the trial for ensuring the guiding principles of trial protocol with regular evaluation of data every alternate week during the trial. The IRB is independent of the study and does not have any competing interests. The trial steering group and the IDMC will conduct regular meetings every fortnight to review the conduct throughout the trial period for auditing.

### Patient and public involvement

No patient and public were involved during the design of the present trial.

### Protocol amendments

Any changes to the protocol will be updated in the clinical trial registry. Afterwards, the principal investigator will notify the trial centers and a copy of the revised protocol will be added to the investigator site file. Any deviations from the protocol will be fully documented using a breach report form.

### Dissemination

The results of the study will be published in a scientific journal, preferably open access, to reach the scientific community. In addition, the results will be communicated to the hospitals which are part of the study and, thereby, the health professionals working in these hospitals. There are no publication restrictions so that also the broader public will be addressed via postings in media.

### Trial status

Protocol version: 1.3 of December 4, 2023.

Recruitment start (expected): June 15, 2024.

Recruitment finish (expected): July 31, 2024.

### Trial registration

The present trial is registered at ClinicalTrials.gov with identifier No: NCT06155474. Registration date is December 4, 2023.

## Discussion

Children suffering from SAM are usually treated with RUTF; however, literature indicates that the iron content present in RUTF may not be sufficient to elevate the iron levels to the optimal range among them [21]. Considering this and the presence of symptoms of diarrhea, pneumonia, acute gastroenteritis, and acute respiratory infections among SAM children, the present trial aims to evaluate the effect of RUTF plus prebiotics on the management and gut microbiome diversity of SAM children. Malnutrition alters the gut microbiome of the children with the gut microbiota of SAM children less diverse as compared to the healthy ones [30]. However, literature highlights the role of prebiotics in stimulating the growth of specific taxa in the gut of the host conferring benefits to their health [31].

Previous studies clearly demonstrate the difference in the diversity of gut microbiome of healthy and malnourished children. Studies indicate a higher relative abundance of Bacteroides in the gut microbiome of children who suffer from diarrhea as compared to those who have recovered from it [32]. Further, the dominance of firmicutes has also been reported in the gut of the healthy host [33]. This is because children who encounter diarrhea at an early age might get impairment in the composition of their gut microbiota, resulting in continuous diarrhea, persistent illness, and underdevelopment [34], whereas GOS, as form of prebiotics, is proven to have positive effects on the growth of beneficial bacteria (including bifidobacterial in the gut), stool consistency, bowel function and transit time, support of natural defenses, and mineral absorption [35, 36]. This indicates that GOS could also provide beneficial results for treating the malnutrition among children by altering their gut microbiome. Likewise, previous trials reveal the beneficial effects of prebiotics on the health of SAM children [22, 37]. However, there is rarely any study that evaluates the role of prebiotics on the gut microbiome of SAM children, to the best of our knowledge.

The present trial is designed in a way that addresses this gap, reduces any potential biases, and pinpoints some major aspects of malnutrition among children that were lacked in the previous trials. However, there are some limitations that should be highlighted. First, the present trial is designed to be conducted in a single district, i.e., Lahore, and may not incorporate regional disparities. Secondly, the present study will evaluate the role of different treatment modalities on the gut microbiome of the children at the end of the trial and does not consider the relapse rate due to time and financial constraints, as the present study is not supported by any external funding sources. Furthermore, the data related to maternal characteristics will be recorded on verbal history which could potentially result in recall or observation bias. However, in order to overcome this, we are currently working to standardize the trial’s procedure and may also take maternal stool samples for analyzing the role of maternal gut microbiome on the child’s health.

## Conclusions

Conclusively, the results of the present trial are expected to indicate RUTF plus prebiotic as a cost-effective therapy for improving the gut microbiome diversity of SAM children and as a more effective therapy for treating SAM children as compared to RUTF individually. GOS present in prebiotics is proven to have positive effects on growth of bifidobacterial, stool consistency, bowel function and transit time, support of natural defenses, and mineral absorption that could also provide beneficial results for treating the malnutrition among children. This is the first trial that evaluates the effect of prebiotics on the gut microbiome diversity of SAM children, as per our knowledge. Despite of its limitations, the results of the present trial would provide useful insights and high-quality data for the treatment and management of SAM children by evaluating the effect of RUTF plus prebiotic on the gut microbiome diversity of children, leading to medical evidence for designing large-scale studies.

## Data Availability

The datasets analyzed during the current study and statistical code are available from the corresponding author on reasonable request, as is the full protocol.

## Abbreviations

CI: Confidence interval
DHQ: District Health Quarter
GOS: Galactooligosaccharides
IDMC: Independent Data Monitoring Committee
IQR: Inter-quartile range
IRB: Institutional Review Board
IRMNCH: Integrated Reproductive Maternal Newborn, Child Health
ITT: Intention-to-treat
MDAT: Malawi Development Assessment Tool
MUAC: Mid-upper-arm circumference
OUT: Operational taxonomic unit
RDP: Ribosomal Database Project
RUTF: Ready-to-use therapeutic food
S: Svedberg
SAM: Severe acute malnutrition
SD: Standard deviation
SPSS: Statistical Package for Social Science
WHO: World Health Organization
